# Sensory Acceptability of Infant Cereals with Whole Grain in Infants and Young Children

**DOI:** 10.3390/nu9010065

**Published:** 2017-01-13

**Authors:** Juan Francisco Haro-Vicente, Maria Jose Bernal-Cava, Amparo Lopez-Fernandez, Gaspar Ros-Berruezo, Stefan Bodenstab, Luis Manuel Sanchez-Siles

**Affiliations:** 1 Department of Research and Development, Hero Group, Alcantarilla, Murcia 30820, Spain; jfrancisco.haro@hero.es; 2 Department of Food Science and Nutrition, University of Murcia, Campus de Espinardo, Espinardo, Murcia 30071, Spain; amparolf@um.es (A.L.-F.); gros@um.es (G.R.-B.); 3 Department of Research and Development, Hero Group, Lenzburg 5600, Switzerland; stefan.bodenstab@hero.ch (S.B.); luisma.sanchez@hero.es (L.M.S.-S.)

**Keywords:** acceptability, complementary feeding, infant cereals, whole grain

## Abstract

In many countries, infant cereals are one of the first foods introduced during the complementary feeding stage. These cereals are usually made with refined cereal flours, even though several health benefits have been linked to the intake of whole grain cereals. Prior evidence suggests that food preferences are developed at early stages of life, and may persist in later childhood and adulthood. Our aim was to test whether an infant cereal with 30% of whole grain was similarly accepted both by parents and infants in comparison to a similar cereal made from refined flour. A total of 81 infants between 4 and 24 months old were included in the study. Parent-infant pairs participated in an 8-day experimental study. Acceptance was rated on hedonic scales (4-points for infants and 7-points for parents). Other attributes like color, smell, and taste were evaluated by the parents. Acceptability for infant cereals with whole grain and refined cereals was very similar both for infants (2.30 ± 0.12 and 2.32 ± 0.11, *p* = 0.606) and parents (6.1 ± 0.8 and 6.0 ± 0.9, *p* = 0.494). Therefore, our findings show that there is an opportunity to introduce whole grain cereals to infants, including those who are already used to consuming refined infant cereals, thereby accelerating the exposure of whole grain in early life.

## 1. Introduction

Scholars have long established that the complementary feeding stage is very important because of the continued rapid growth and many changes that affect children’s health and development [1,2,3]. These changes have a major influence on nutritional status during infancy and the food preferences during childhood and adulthood [4,5,6]. For this reason, several authors have strongly recommended the intake of healthy foods from the very beginning of the complementary feeding stage [7,8,9].

Weaning practices are significantly influenced by cultural beliefs [10,11]. However, in many countries, infant cereals are one of the first foods introduced at the beginning of the complementary period [11,12,13,14]. One possible reason stems from their sensorial and digestive properties [15]. For millennia, cereals have been staples for humans and are currently a large part of U.S. Dietary Guidelines [16]. Cereals are a good source of energy and macronutrients, such as carbohydrates, proteins and fats, that are needed for growth. Cereals are also an important source of vitamins, minerals and other essential bioactive compounds necessary for health [17].

Cereals can be consumed as whole grain or refined. Although the definition of whole grain is currently under discussion, the International American Association of Cereal Chemistry (AACC) defined whole grains as “intact, ground, cracked or flaked caryopsis, whose principal anatomical components—the starchy endosperm, germ, and bran—are present in the same relative proportions as they exist in the intact caryopsis” [18]. In 2010, the Healthgrain Consortium broadened the AACC definition of whole grain to include small losses of components—that is, less than 2% of the grain/10% of the bran. Such losses may occur through processing methods required to ensure the safety and quality of whole grain and are allowed under the above definition [19].

It should be taken into account that until the 19th century, cereals were consumed as whole grain; it was only during the Industrial Revolution with the advent of new milling techniques that the bran and germ were removed from the grain kernel, obtaining refined cereal flours with improved texture and taste and ultimately leading to longer shelf life [20].

Several organizations and scholars have acknowledged the benefits associated with the intake of whole grain cereals for adults and children above two years [21,22,23]. Moreover, the USDA (US Department of Agriculture) recommends that, during the second semester of life, infants should be gradually introduced to fiber-containing foods, such as whole grain cereals, vegetables, and legumes [10]. Although there is no general agreement regarding daily dietary intake recommendations for whole grain (even for adults) [23,24], these recommendations are important and should be further studied in infants and young children. As eating habits can be molded during complementary feeding time, the early introduction and consumption of infant cereals elaborated with whole grain flour could be desirable [22].

In most countries, commercial infant cereals are made with refined cereal flours (www.innovadatabase.com). One of the main problems found when whole grain is introduced into the diet of an adult is the low sensorial acceptability compared to refined cereal-based foods [23,25]. However, prior research reveals that infants and young children have a higher acceptance of new foods in the complementary feeding period until the age of 18–24 months [26,27,28]. In this vein, several authors recommend gradually introducing whole grain products by substituting refined cereals as an effective way of incorporating whole grain into consumers’ diets [23]. This can be reinforced by the development of technological processes to improve sensorial properties of whole grain products [29,30].

The present study was designed to determine if the intake of an infant cereal-based product with 30% whole grain was similarly accepted both by parents and their children, compared to the same infant cereal without whole grain (refined cereals).

## 2. Materials and Methods

### 2.1. Infant Cereal Samples

All infant cereals used in this experiment were commercially available products from HERO ESPAÑA S.A (Murcia, Spain). We selected this brand of multicereals to conduct this study because it is one of the most widely consumed infant cereals on the Spanish market. One of two infant cereals contained 30% of whole grain flour, WGC (new recipe), and the other one 100% refined cereal flours, RC (old recipe). The ingredients for WGC were: hydrolyzed cereal flour (wheat, wheat whole grain, corn, rice, oat, barley, rye, sorghum and millet), minerals, natural flavor and vitamins where the content of wheat whole grain flour was 30% of total cereals. The ingredients of RC were: hydrolyzed cereal flours (wheat, corn, rice, oat, barley, rye, sorghum and millet), minerals, natural flavor and vitamins. The nutritional composition of the two infant cereals used in this study is described in Table 1.

### 2.2. Participant Characteristics

There were 81 parents with infants between the ages of 4–24 months recruited through advertisement on the website of the University of Murcia (Spain), as well as in kindergartens and parents’ circles. Eligible healthy infants had a gestational age of 37–42 weeks and a birth weight greater than 2500 g and had been fed with gluten-containing cereals prior to enrolment in the experiment. Infants that had food allergies, swallowing or digestion problems, or other medical issues that could influence the ability to eat were excluded. There were an additional 20 pairs excluded because the parents did not complete the study or did not return the questionnaire (See Supplementary Materials). Once all parent-infant pairs were recruited, they were assigned to only one group where they received two packages of infant cereals (WGC and RC). The distribution of samples was counterbalanced to avoid any possible bias. The study protocol was approved by the Research Ethical Committee of the University of Murcia. Written informed consent was obtained from both parents of all participating infants before the inclusion.

### 2.3. Data Collection

Testing was carried out at home during a period of eight days. For the home-use test, the parents were responsible for conducting the experiment and collecting the data requested in the questionnaire (See Supplementary Materials). Prior to testing, the parents received both detailed written and oral instructions.

#### 2.3.1. Testing

Parents received two coded packs of cereals. During days 1 and 2, infants were accustomed to the first of the two cereals. On the third day, parents evaluated the acceptance. Over days 4 and 5, infants continued eating the cereal which they used to eat before enrollment in the present study. On day 6, the same process started with the second infant cereal—familiarization on days 6 and 7 and, on day 8, evaluation of acceptance. No additional foods or beverages were introduced into the infants’ diet during the study. All samples used in this study were packaged into identical foil bags. Each bag was marked with a three-digit randomization code. At the moment of feeding, the samples were counterbalanced and randomized; consequently, a child was fed with one sample on one day and the other sample on the other day. Each parent fed his/her infant in the habitual place, at a normal pace until the infant refused the spoon or bottle three consecutive times. Rejection behaviors are typically turning the head away, closing the mouth firmly, pushing the spoon away, spitting the food out or becoming upset [14,31]. Before each feeding, it was necessary to ensure that the infant was sufficiently hungry. In particular, parents were asked not to feed their infants with infant milk, other beverages, or solid foods for 1 h before the cereal intake [14,32]. Furthermore, testing occurred at approximately the same time of the day and 30 to 60 min before the infants’ next scheduled feeding, so that variation of intake was not affected by different levels of hunger or satiation, but rather reflected hedonic response to the food. Regarding mode of preparation, this depended on the parents’ habits (using bottle or spoon). Parents performed the sensory evaluation of the same products only after feeding their infants to ensure no interference with their infant’s reactions due to non-habitual parent behavior (product testing) during the feeding (days 3 and 8) [14,32].

#### 2.3.2. Measure of Food Acceptance

Parents were first asked to answer questions about their infants, feeding practices as well as socio-demographic information, such as gender, age and education (See Supplementary Materials). Then, parents were asked to score their degree of liking using a 7-point hedonic scale ranging from 1 “dislikes very much” to 7 “likes very much.” This test allowed us to evaluate the acceptability of each parent for the sample of cereal. Parents’ liking represents one important step to deciding if this type of cereal would be suitable for their infants. For the assessment of the acceptance by children, we used a 4-point hedonic scale [14,31]. This scale uses the following scores: ”−− “ (very negative) if the infant spit out the food, frowned, pushed the spoon away or stopped eating; “−“ (negative) if the infant ate a couple of spoonful, grimaced and stopped eating; “+” (positive) if the infant ate some of the food without a specific reaction; “++” (very positive) if the infant accepted the first spoonful immediately and displayed signs of content, such as a relaxed face or a smile [14,31].

### 2.4. Statistical Analyses

Data are expressed as means ± standard error of mean (SEM) for the following variables: age of introduction of infant cereals and parent’s perception of child’s degree of liking. For variables related to sensory analysis, data were expressed as mean ± standard deviation (SD). The categorical variables were expressed as percentage (%).

Descriptive analysis was employed to describe parents’ and infants’ characteristics as well as all variables related to consumption, mode of preparation, frequency of intake of infant cereals and sensory analysis. The evaluation of infants’ acceptance, assessed by parents, was converted into scores of −3, −1, 1 and 3 so that there were equal intervals between adjacent scores throughout the scale. Taking into account that all variables are not distributed normally (Kolmogorov–Smirnov test), non-parametric analyses were performed: the Wilcoxon test for paired samples was used to detect differences in sensorial variables between the two samples of infant cereals; in the case of cereal feeding practices, variables expressed as percentages as per the Mann–Whitney test were applied in order to detect difference between both age ranges.

All results with a significance level of *p* < 0.05 were considered statistically significant. Statistical analyses of data were performed using the Statistical Package for the Social Sciences (SPSS version 19.0; Inc., Chicago, IL, USA).

## 3. Results

### 3.1. Subject Characteristics and Cereal Feeding Practices

The characteristics of the 81parent-infant pairs are presented in Table 2. The mean age of introduction of infant cereals was 5.2 (±1.3) months. The type of milk used by parents to prepare the cereals was in 55.6% of cases as follows: 21.0% “growing-up” milk; 13.5% cow’s milk; 7.4% infant formula; and 2.5% breast milk (Table 3).

Parents were asked about the cereal feeding mode (bottle and/or spoon) and the number of times infants were fed per day (Table 3). The cereal feeding mode was distributed as follows: most infants (70.4%) were taking cereals from a bottle, 16.0% took cereals with a spoon and 13.6% used both bottle and spoon. There were significant differences in the mode of preparation of infant cereals depending on the age group (*p* < 0.05). Cereal bottle feeding was higher in the group of infants between 13 and 24 months of age (80%) as compared to the group of infants below one year. In general, more than 50% of infants took two servings a day, almost 40% took one serving a day, and the rest between three or more servings (7.4%) without significant differences between the two age ranges studied (below and above one year of age) (Table 3).

**Figure 1:**
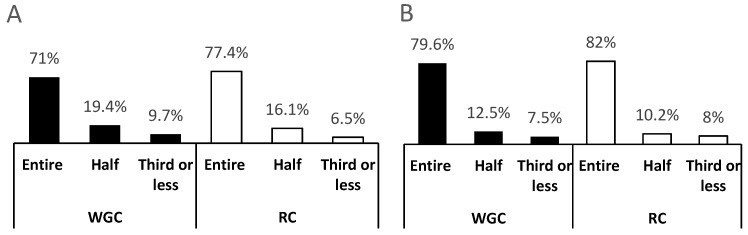
Infants’ consumption of the serving of infant cereals prepared by parents. WGC: Whole Grain Cereal; RC: Refined Cereal. Values are expressed as percentage of total infants. (**A**) represents the consumption of cereals by infants 4–11 months of age; (**B**) consumption of cereals by infants 12–24 months of age.

The average amount of WGC and RC cereals consumed by age group is shown in Figure 1. In both cases, over 75% of infants consumed the entire serving of the cereals prepared, with no significant differences between the infant cereals evaluated.

### 3.2. Parents’ Perception of Infants’ Liking

As shown in Table 4, parents’ rating of their infants’ degree of liking was not statistically significant between the two infant cereals evaluated (WGC and RC)—neither within the two age groups (*p* = 0.317, *p* = 0.666 for group 4–11 months and group 12–24 months, respectively) nor in the total sample (*n* = 81, *p* = 0.606). Both infant cereals were highly accepted with average scores higher than 2.

### 3.3. Parents’ Acceptability of Infant Cereals

There were no significant differences between WGC and RC in any of the attributes, as shown in Table 5. Parental rating of liking for each attribute did not differ for any infant cereal, indicating that parents reported the same liking for each sample. In general, the score for each attribute was very similar between both samples and overall acceptability was rated very high for both cereal products (6.1 for WGC and 6.0 for RC). In addition, we asked parents why they liked the infant cereals and which attributes influenced their choices. In both cases, the criteria for their choices were similar; for example, about 50% of the parents chose the taste as first choice, followed by aroma (20%), and texture (17%).

## 4. Discussion

The present study reveals that the addition of whole grain in infant cereals was similarly well accepted from the sensorial point of view by both parents and their infants as compared to refined cereals. Importantly, previous studies have observed that early exposure to foods affects infants’ later taste acceptance patterns and that infants are able to communicate their acceptance by both quantity of intake and facial expression [32]. In our study, we did not observe any differences in the facial expression or in the intake, and more than 75% of the infants ate the entire cereal serving prepared by their parents. Several factors are likely to explain the high acceptability found in whole grain infant cereals such as the percentage of whole grain used in this study (30%), the milk used for the reconstitution of the cereal (already accepted by the infants) or technology advances in cereal processing which improve the sensorial characteristics of whole grain cereals.

The selected amount of whole grain tested in our study was based on the minimal amount of whole grain with possible beneficial effects. Although there is no defined adequate intake of whole grain for adults or infants, Ferruzi et al. [15] have suggested that a whole grain food should provide 8 g of whole grain per 30 g serving (27 g/100 g) in order to be defined as a whole grain food which is nutritionally meaningful.

Interestingly, it has been reported that the main factor of consumer rejection in whole grain-based products is its bitter taste and rough texture [25,33]. In our study, we did not observe any of these issues. In fact, parents were reporting similar sensory liking scores in taste and texture and high scores in both infant cereals. Our results are similar to those found in students by Magalis et al. (2016), who did not find statistical differences in bitter taste between refined and whole grain products, although sensorial preferences tended toward some refined cereal products [25]. Other sensorial studies in school children have also reported a similar degree of acceptability of whole grain versus refined cereal products, i.e., whole grain products were well accepted [34,35].

Whole grain cereals, as well as vegetables, can be bitter due to their content of polyphenolic compounds [36]. Similar to findings in our study, Lange et al. (2013) and Mennella et al. (2015) reported that when the new foods are introduced (vegetables) with other foods already accepted, the acceptability of new foods was better [14,37]. This effect was related to the reduction of the unpleasant, bitter or sour notes of the vegetables not only by dilution, but also by the sweetness of the milk. The sweetness of breast milk has been estimated as equivalent to a 2.12% solution of sucrose [38]. The sweetness of standard formula milks is similar [39]. Salty and sweet tastes have the characteristic of blocking or masking unpleasant tastes present in many foods, such as a bitter taste in vegetables [38,39]. A clinical study of school-aged children has shown that the presence of diluted solutions of a sweetener (aspartame) along with vegetables increased the liking of vegetables and decreased the perception of bitter taste [40]. For that reason, we could hypothesize that like in vegetables, our infant cereals with whole grain were highly accepted due to the fact that they were reconstituted with infant milk formulas or breast milk previously accepted by the infant. In our study, whole grain cereals were mainly prepared with milk formula and to a lesser extent with breast milk. The presence of sweet taste and its acceptance by infants could mask the bitter notes present in the whole grain cereals, leading the products to be accepted in the same way as the refined product [41,42].

Regarding infant cereal practices, our study shows that the mean age of introduction of cereals was 5.2 months. In Spain, infant cereals are usually the first food introduced at the beginning of weaning and therefore the age of introduction in our study was in line with current recommendations of first introducing products between 4 and 6 months of age [3,43].

The complementary feeding period is a “critical time window” in human development during which eating behaviors are developed [44]. For this reason, timing, type and ways the foods are introduced are important feeding practices in the development of acceptance of healthy food as part of a healthy diet. Cereals play a main role at the beginning of and during the complementary feeding period. The type of cereals consumed (whole grain or refined) may influence both health and nutritional status of infants. In adults, it has been shown that significant consumption of whole grain products is associated with a lower risk of cardiovascular diseases, diabetes, obesity, colon cancer and gastrointestinal health [36]. A previous meta-analysis concluded that there is a negative correlation between intake of whole grain and mortality, with a reduction of 7% in risk associated with each single serving/day increase in whole grain intake [45]. Due to increasing evidence of health benefits associated with whole grains, several authors have strongly recommended implementing strategies for educating consumers to gradually incorporate whole grains into their diets through the substitution of refined cereals [23]. Although the beneficial effects of whole grains have not been demonstrated in infants or young children due to the lack of clinical trials in this age range, the development of healthy dietary habits during early stages of infancy including a diet with whole grain products could be desirable [22].

Two potential risks of the use of whole grain in infant cereals should be taken in account. Firstly, as compared to refined rice, whole grain rice has a higher content of inorganic arsenic, which is concentrated in the bran layers [46,47]. Secondly, in unbalanced diets, excessive fiber content is likely to have negative effects on mineral bioavailability [48]. In order to avoid these issues, we have used whole grain wheat in compliance with European infant legislation (Directive 2006/125/CE) [48]. Also, the fiber content of the cereals used in our study was within commercial standard values.

## 5. Conclusions

This research was conducted as a preliminary study toward a clinical trial designed to evaluate whole grain effects in infants with ages ranging from 5 to 9 months. Interestingly, we found that infant cereals with 30% whole grain were very well accepted from a sensory point of view by infants between 4 to 24 months as well as by their parents. Moreover, there is a lack of studies showing that refined cereal flours in infants and young children are better from a nutritional point of view than whole grain infant cereals. Throughout most of human development, whole grain cereals—not refined cereals—were naturally consumed. Therefore, there might be an opportunity to (re)introduce whole grain cereals to infants who are accustomed to consuming refined infant cereals, thereby accelerating the exposure of whole grain in early life. This research represents a first step in our understanding of sensory acceptability of whole grain infant cereals in infants aged 4 to 24 months. We encourage future studies to analyze both the acceptability of higher percentages of whole grain in infant cereal-based products as well as possible imprinting of health effects on infants during the complementary feeding period.

## Figures and Tables

**Table 1:** Nutritional composition of the two infant cereals used in the study.

Mean Value (100 g)	WGC	RC
Energy (kcal)	380	372
Protein (g)	9.1	8.5
Carbohydrates (g)	78	81.6
Sugars (g)	21	22
Fat (g)	2.3	1.3
Fiber (g)	5.2	3

WGC: Whole Grain Cereal and RC: Refined Cereal.

**Table 2:** Characteristics of infants and parents who participated in this study.

Characteristics	Group (*n* = 81)
Infants	
Age (by ranges)	
4–6 months	8.6
7–9 months	13.6
10–11 months	16
12–24 months	61.8
Girls	60
Parents	
Men	12
Women	88
Age	
25–30 years	5.3
31–35 years	46
36–40 years	43.4
>41 years	5.3
Number of children	
One child	48
Two children	48
Three children	4

Values expressed as percentage.

**Table 3:** Cereal feeding practices used for parents to prepare the infant cereals.

	Age Range		
	4–11 Months	12–24 Months	Total
**Type of Milk Used**			
Breast milk	6.5 (2)	-	2.5 (2)
Infant formula	9.7 (3)	6.0 (3)	7.4 (6)
Follow on formula	77.4 (24)	42 (21)	55.6 (45)
Growing up milk	3.2 (1)	32 (16)	27 (17)
Cow’s milk	3.2 (1)	20 (10)	13.6 (11)
**Mode of Cereal Feeding ^a^**			
Bottle	54.8 (17)	80 (40)	70.4 (57)
Spoon	25.8 (8)	10 (5)	16 (13)
Both	19.4 (4)	10 (5)	13.6 (11)
**Frequency of Intake**			
One serving	48.4 (11)	34 (17)	39.5 (32)
Two serving	41.9 (13)	60 (30)	53.1 (43)
Three or more serving	9.7 (3)	6 (3)	7.4 (6)

Values are expressed as percentage of total infant (number of infants); ^a^ Superscript indicates that there is significant differences between both groups of age and mode of feeding cereals (*z* = −2.281; *p* < 0.05).

**Table 4:** Parent’s perception of child’s degree of liking (mean ± SEM).

Age	WGC (*n* = 81)	RC (*n* = 81)	*p*-Value
4–11 months	2.35 ± 0.19	2.16 ± 0.18	0.317
12–24 months	2.29 ± 0.16	2.40 ± 0.15	0.666
Total (*n* = 81)	2.3 ± 0.12	2.32 ± 0.11	0.606

WGC: Whole Grain Cereal; RC: Refined Cereal.

**Table 5:** Sensory evaluation of infant cereals by parents (mean ± SD).

Attributes	WGC	RC	*p*-Value
Color	6.1 ± 0.9	5.8 ± 1.1	0.090
Aroma	6.3 ± 0.9	6.3 ± 0.8	0.850
Taste	6.2 ± 0.9	6.1 ± 0.9	0.799
Texture	5.9 ± 1.1	6.2 ± 1.1	0.235
Overall acceptability	6.1 ± 0.8	6.0 ± 0.9	0.494

* Values in rows with different superscripts are significantly different (*p* < 0.05).

## Supplementary Materials

The following are available online at http://www.mdpi.com/2072-6643/9/1/65/s1. Data Collection Questionnaire used in study.
