# Supplementary material for: Sensory Acceptability of Infant Cereals with Whole Grain in Infants and Young Children
Source: Nutrients. 2017 Jan 13;9(1):65. doi: 10.3390/nu9010065 (PMC5295109; doi:10.3390/nu9010065)
Supplement: Supplementary file 1 [file nutrients-09-00065-s001.docx]

Supplementary Materials: Sensory Acceptability of Infant Cereals with Whole Grain in Infants and Young Children

Juan Francisco Haro-Vicente, Maria Jose Bernal-Cava, Amparo Lopez-Fernandez,
Gaspar Ros-Berruezo, Stefan Bodenstab and Luis Manuel Sanchez-Siles

Acceptability Survey

INFANT CEREALS, “MULTICEREALS”

1. OBJECTIVE

To determine if one or more noticeable sensory differences among several samples are present, evaluating in the order of intensity and acceptability.

1. WHICH TYPE OF PRODUCTS WILL BE TASTED?

Parents have been given two bags of infant cereals, “Multicereals,” in duplicate along with a survey for evaluating the products. Each one of the cereal bags is encoded with a three-digit number. One sample is in the standard range whereas the other one contains whole grain.

1. HOW SHOULD YOU FILL IN THIS SURVEY?
   1. As inclusion criteria, only parent/infant pairs who are healthy and without health problems or illness (i.e., celiac disease) can participate.
   2. At the beginning of this survey, you will find a set of questions related to the consumption habits of your son/daughter, e.g., when he/she started on infant cereal, how it is served, etc.
   3. Most of the questions must be assessed by yourself according to your opinion, but there are others that must be assessed according to the reaction of your baby.
   4. The tasting will be at home in the place where your child is usually fed. Mother or father must carefully follow the instructions on how to assess your child’s reactions.
   5. If, for any reason, you couldn’t give your child infant cereal at the appointed time, you can try another time of day or on subsequent days.

PERSONAL DATA AND FEEDING HABITS

1. This questionnaire is filled in by: Mother Father

1. Age (Mother or Father):

1. Could you indicate your marital status? Married Single Live as a couple
2. What is the highest educational level you have achieved?

| No education |  |
| --- | --- |
| Primary education |  |
| Secondary education |  |
| University degree |  |

1. Currently, who provides your household income? One….. Both….. None…..
2. Could you tell us how many children you have? 1….. 2….. 3….. ≥4 children…..

1. About the child or children who will go through the tasting, could you indicate his/her age:

| Age (Months) | 1st baby | 2nd baby | 3rd baby | 4th baby |
| --- | --- | --- | --- | --- |
| 3–6 |  |  |  |  |
| 7–9 |  |  |  |  |
| 10–11 |  |  |  |  |
| 12–24 |  |  |  |  |
| >24 |  |  |  |  |

1. What is the gender of your child? Boy….. Girl…..
2. What is his/her current weight body? _________ grams
3. What kind of milk do you give your child?

Breast milk….. Starter milk….. Follow-on formula….. Growing-up milk…..

Cow’s milk….. Mix…..

1. How old was your child when you completely stopped breastfeeding?

| Months | | | | | | | | | | | | | |
| --- | --- | --- | --- | --- | --- | --- | --- | --- | --- | --- | --- | --- | --- |
| 1 | 2 | 3 | 4 | 5 | 6 | 7 | 8 | 9 | 10 | 11 | 12 | 13 | 14 |
|  |  |  |  |  |  |  |  |  |  |  |  |  |  |

1. At which age did your baby start eating?

|  | Months | | | | | | | | | |
| --- | --- | --- | --- | --- | --- | --- | --- | --- | --- | --- |
|  | 3 | 4 | 5 | 6 | 7 | 8 | 9 | 10 | 11 | 12 |
| Infant cereals without gluten |  |  |  |  |  |  |  |  |  |  |
| Infant cereals with gluten |  |  |  |  |  |  |  |  |  |  |
| Vegetable jars |  |  |  |  |  |  |  |  |  |  |
| Fruit jars |  |  |  |  |  |  |  |  |  |  |
| Fish jars |  |  |  |  |  |  |  |  |  |  |
| Meat jars |  |  |  |  |  |  |  |  |  |  |

1. Are you currently giving your baby infant cereal? Yes……. No…….
2. What kind of infant cereals do you give your child?

Milky cereals….. Non-milky cereals….. Both…..

1. In the case of giving your child milky cereals, could you tell us your reasons? (open-ended question)
2. Currently, could you tell us which brand of cereal you are using?
3. In the case of serving non-milky cereals, how is it prepared?

Mother’s milk….... Starter formula..….. Follow-on formula……..

Cow’s milk…..…. Growing-up milk..……. Water……... Other……..(specified)

0

1. If preparing non-milky cereals with infant formula, which brand of infant formula are you currently using? (open-ended question)
2. How many times do you give your child infant cereal per day?

1….. 2 ….. 3….. ≥4…..

1. How often do you give your child infant cereal per week?

Daily……. Less than 3 times per week……. More than 3 times per week…….

1. At what time of day and how do you give your child the infant cereals? (Put an X in the appropriate box)

|  | Baby Bottle | Bowl | Both |
| --- | --- | --- | --- |
| Breakfast |  |  |  |
| Lunch |  |  |  |
| Snack |  |  |  |
| Dinner |  |  |  |

1. When preparing infant cereals (in bowl/bottle), could you tell us which recommendations you follow?

Pediatrician

Friends/Relatives

My own criteria

Label of product

Other (please indicate) ( )

1. What kind of utensil do you use to measure the cereal and the number of servings? (You must first mark the kind of tool in the box and then the number of servings.)

|  |  | Number of serving | | | | | | | | | |
| --- | --- | --- | --- | --- | --- | --- | --- | --- | --- | --- | --- |
|  |  | 1 | 2 | 3 | 4 | 5 | 6 | 7 | 8 | 9 | 10 |
| The scoop provided by the milk powder |  |  |  |  |  |  |  |  |  |  |  |
| A tablespoon |  |  |  |  |  |  |  |  |  |  |  |
| A teaspoon |  |  |  |  |  |  |  |  |  |  |  |
| Added directly from bag until thickened |  |  |  |  |  |  |  |  |  |  |  |

DAY 3. TASTING MOTHER/FATHER

Beginning with the sample encoded with the number “XXX” you must prepare the baby cereal either in a bowl or bottle as you normally do every day. Then you must try it and answer a set of questions related to the product you are tasting. If you prepare a bottle, you can try it directly from a glass.

1. Evaluating the product as “OVERALL IMPRESSION,” how much do you like or dislike this product?

| Like very much |  |
| --- | --- |
| Like moderately |  |
| Like slightly |  |
| Neither like nor dislike |  |
| Dislike slightly |  |
| Dislike moderately |  |
| Dislike very much |  |
|  |  |

1. Could you tell us the reasons why you LIKED this product? Reply if you liked the product
2. Could you tell us the reasons why you DISLIKED this product? Reply if you disliked the product

|  | LIKE VERY MUCH | LIKE MODERATELY | LIKE SLIGHTLY | NEITHER LIKE NOR DISLIKE | DISLIKE SLIGHTLY | DISLIKE MODERATELY | DISLIKE VERY MUCH |
| --- | --- | --- | --- | --- | --- | --- | --- |
| COLOR |  |  |  |  |  |  |  |
| SMELL |  |  |  |  |  |  |  |
| TASTE |  |  |  |  |  |  |  |
| TEXTURE (only in case of bowl) |  |  |  |  |  |  |  |

1. Assess each one of the sensory attributes using the scale indicated below:

DAY 3. TASTING BABY

Once you have assessed baby cereals with code “XXX” you must prepare one for your baby as you normally do every day. Then you must assess the reactions your baby has when trying the cereal. To carry out the test of acceptability by your child, you must remember several points when proceeding with the tasting:

- Avoid facial expressions that can distract your child.
- The tasting ends when your child **rejected the spoon more than three successive times*.***
- Your child must not eat anything one hour before the tasting.
- It is important to maintain the same hour of feeding each day

1. Mark the answer that most resembles the reaction of your son/daughter at the time of testing the baby cereal:

“− −“ **(very negative); if the infant spat out the food, frowned, pushed the spoon away or stopped eating**

“−“ (negative); **if the infant ate a couple of spoonful, grimaced and stopped eating;**

“+” (positive); **if the infant ate some of the food without a specific reaction**

“+ +” (very positive); **if the infant accepted the first spoonful immediately and displayed signs of content, such as a relaxed face or a smile.**

1. Could you tell us the amount of cereal (in bowl or bottle) that your baby has taken:

| He/she ate/drank all |  |
| --- | --- |
| He/she ate/drank half |  |
| He/she ate/drank one-third |  |
| He/she ate/drank three-quarters |  |
| He/she ate/drank something (4–6 spoons) |  |
| Did not eat/drink anything |  |
| Others (please indicate quantity) |  |

1. If you have any additional comments about the tasting session, please include them here:

DAY 8. MOTHER/FATHER’S TASTING

You will proceed the same way as the day before, but in this case you will taste baby cereals with the code “YYY”. First, you must prepare baby cereal in a bowl or bottle and then try it and answer a set of questions related to this product.

1. Evaluating the product as “OVERALL IMPRESSION,” how much did you like or dislike this product?

| Like very much |  |
| --- | --- |
| Like moderately |  |
| Like slightly |  |
| Neither like nor dislike |  |
| Dislike slightly |  |
| Dislike moderately |  |
| Dislike very much |  |
|  |  |

1. Could you tell us the reasons why you LIKED this product? Reply if you liked the product
2. Could you tell us the reasons why you DISLIKED this product? Reply if you disliked the product

|  | LIKE VERY MUCH | LIKE MODERATELY | LIKE SLIGHTLY | NEITHER LIKE NOR DISLIKE | DISLIKE SLIGHTLY | DISLIKE MODERATELY | DISLIKE VERY MUCH |
| --- | --- | --- | --- | --- | --- | --- | --- |
| COLOR |  |  |  |  |  |  |  |
| SMELL |  |  |  |  |  |  |  |
| TASTE |  |  |  |  |  |  |  |
| TEXTURE (only in case of bowl) |  |  |  |  |  |  |  |

1. Assess each one of the sensory attributes using the scale indicated below:

DAY 8. BABY’S TASTING

Once you have assessed baby cereals with code “YYY” you must prepare one for your baby as you normally do every day. Then you must assess the reactions your baby has when trying the cereal. To carry out your child’s test of acceptability, you must remember several points when proceeding with the tasting:

- Avoid facial expressions that can distract your child.
- The tasting ends when your child **rejects the spoon more than three successive times*.***
- Your child must not eat anything one hour before the tasting
- It is important to maintain the same hour of feeding each day

1. Mark the answer that most resembles the reaction of your son/daughter at the time of testing the baby cereal:

“− −“ **(very negative); if the infant spat out the food, frowned, pushed the spoon away or stopped eating**

“−“ (negative); **if the infant ate a couple of spoonful, grimaced and stopped eating;**

“+” (positive); **if the infant ate some of the food without a specific reaction**

“+ +”(very positive); **if the infant accepted the first spoonful immediately and displayed signs of content, such as a relaxed face or a smile.**

1. Could you tell us the amount of cereal (in bowl or bottle) that your baby has taken:

| He/she ate/drank all |  |
| --- | --- |
| He/she ate/drank half |  |
| He/she ate/drank a third |  |
| He/she ate/drank three-quarters |  |
| He/she ate/drank something (4–6 spoons) |  |
| Did not eat/drink anything |  |
|  |  |

1. If you have any additional comments about the tasting session, please include them here:

MANY THANKS FOR YOUR COLLABORATION
